# Psychological Interventions for Survivors of Intimate Partner Violence in Humanitarian Settings: An Overview of the Evidence and Implementation Considerations

**DOI:** 10.3390/ijerph19052916

**Published:** 2022-03-02

**Authors:** Daniel P. Lakin, Claudia García-Moreno, Elisabeth Roesch

**Affiliations:** 1 Department of Mental Health, Johns Hopkins Bloomberg School of Public Health, 624 N Broadway, Baltimore, MD 21205, USA; 2 Department of Sexual and Reproductive Health, World Health Organization, 20 Ave Appia, 1227 Geneva, Switzerland; garciamorenoc@who.int; 3 Independent Researcher, 5000 MacArthur Blvd #9513, Oakland, CA 94613, USA; elisroesch@gmail.com

**Keywords:** gender-based violence, mental health, psychological interventions, humanitarian, intimate partner violence, sexual violence

## Abstract

This paper provides an analytical overview of different types of psychological interventions that have demonstrated efficacy in low-income and/or humanitarian settings and points to special considerations that may be needed if used with women who have been subjected to gender-based violence (GBV). This paper reviews diverse therapeutic modalities and contrasts them across several domains, including their conventional use and principles; their documented use and efficacy in humanitarian settings; any special considerations or modifications necessary for GBV-affected clients; and any additional resources or implementation concerns when working in low-income contexts. By examining the evidence base of multiple interventions, we hope to provide clinicians and GBV-prevention advocates with an overview of tools/approaches to provide survivor-centered, trauma-informed responses to GBV survivors. This analysis responds to the growing recognition that gender-based violence, in particular intimate partner violence and sexual violence, is strongly associated with mental health problems, including anxiety, depression, and post-traumatic stress. This is likely to be exacerbated in humanitarian contexts, where people often experience multiple and intersecting traumatic experiences. The need for mental health services in these settings is increasingly recognized, and a growing number of psychological interventions have been shown to be effective when delivered by lay providers and in humanitarian settings.

## 1. Introduction

Gender-based violence (GBV) has been shown to have profound, negative impacts on psychosocial and mental health, and presents a global challenge to gender equality, equity and safety. Women who experience GBV can face rejection from their families and communities, experience stigma and be exposed to ongoing risks of violence [1]. GBV survivors also experience higher rates of mental disorders such as depression [2], post-traumatic stress (PTS) [3], and anxiety disorders [4], and are more likely to have attempted suicide. There also appears to be a bidirectional relationship between GBV and mental health problems, in that experiencing GBV seems to increase the likelihood of developing mental health problems, while mental health problems increase the risk of experiencing GBV.

Globally, nearly one in three women (30%) will experience physical and/or sexual intimate partner violence (IPV) or sexual violence by a non-partner in their lifetime [5] and the risk of GBV often grows more acute in humanitarian contexts [6]. A study from South Sudan indicated that up to 65% of women there reported experiencing intimate partner and sexual violence [7], and countries affected by conflict were among those with the highest prevalence of IPV [5]. Humanitarian contexts also pose a particular challenge for addressing the mental health needs of survivors, as health systems can be severely compromised, mental health services may be lacking or non-existent, and access barriers, particularly due to displacement and insecurity, can increase. 

There is evidence that adapted, culturally-relevant mental health programming is an effective tool for addressing poor mental health in humanitarian contexts [8,9,10,11], and research related to mental health interventions for GBV survivors is promising albeit limited. There is some evidence that mental health interventions can reduce the impact of intimate partner violence in humanitarian contexts [12], and that involvement in mental health interventions can improve outcomes related to depression, PTS, and substance use problems [13,14], though there are limited numbers of high-quality studies available [14]. Psychological interventions such as cognitive behavioral therapy, acceptance and commitment therapy, eye movement desensitization and reprocessing (EMDR), and the common elements treatment approach (CETA) have been implemented in humanitarian settings. Moreover, there is a growing body of evidence to suggest that interventions conducted by paraprofessionals and trained lay providers can be effective for common mental disorders [15]. This is particularly important in light of the limited resources (human, financial and organizational) available for mental health in most countries and particularly in low- and middle-income contexts [16].

In addition to questions about effectiveness, questions also remain about what types of interventions are best suited to GBV survivors, whether GBV survivors need tailored interventions and whether existing interventions may be problematic if applied without attention to the particular dynamics of GBV, particularly intimate partner/domestic violence where the violence often is ongoing. Mental health problems for GBV survivors occur within a social and cultural context of gender inequality, discrimination, normalization or acceptance of violence against women, and stigma. This broader context shapes the mental health outcomes of survivors and, we posit, should be considered by health and mental health providers when working with this specific population. The purpose of this review is therefore to identify effective mental health interventions that have been used for survivors of GBV, explore their relative strengths and limitations, and list any potential considerations specific to working in humanitarian contexts. Our goal is to provide an overview of potential interventions and their implementation considerations for clinicians and GBV programming advocates to refer to when developing a combined GBV and mental health program. 

In this we review, we identify and discuss a range of psychological interventions that have demonstrated efficacy in low-income and humanitarian settings and point to special considerations that may be needed if used with women who have experienced GBV in humanitarian or resource-poor settings. We reviewed numerous therapeutic modalities and contrasted them across several domains, including their conventional use and principles; their documented use and efficacy in humanitarian settings; any special considerations or modifications necessary for GBV-affected clients; and any additional resources or implementation concerns when working in low-income contexts. By examining the evidence base of multiple interventions, we hope to provide clinicians and GBV-prevention advocates with an overview of tools/approaches to provide survivor-centered, trauma-informed responses to GBV survivors. 

## 2. Methods and Search Strategy

The original purpose of this review was to inform a manual for clinicians and GBV advocates. This review was intended as a reference for potential interventions to consider when including mental health services in novel GBV intervention programs and vice versa.

The articles, books and chapters consulted in this desk review were identified across PubMed, Google Scholar, and PsycINFO. We included peer-reviewed, published articles, published book chapters, and reports from non-governmental organizations related to the development and testing of mental health and psychosocial services programming in low-income and humanitarian contexts. The search included three phases taking place in May 2020. The first phase identified well-established mental health interventions with at least one trial in a humanitarian context. Our search strategy included all articles published within the last 20 years. Trials must have been conducted in a low-income or humanitarian context. The type of mental health intervention used, effect sizes, whether the sample was GBV affected, and what, if any, adaptations were made to the intervention were all noted. Articles were excluded if: (1) they were published before the year 2000; (2) if the study design was not either a randomized-clinical trial or in a comparative group format; (3) if the study was not conducted in a humanitarian or low-income context; (4) if the article was not peer reviewed; (5) if the sample included children; and (6) if the investigation was not conducted in a low- or middle-income context. The search terms used for this initial phase are included in Appendix A. 

The second phase involved consulting clinical guidebooks and peer-reviewed literature that explained the principles and guiding practices of each individual intervention identified in the previous phase. The purpose of this phase was to identify and explore the concrete principles required for meaningful clinical implementation of a given intervention, and explore its feasibility in a resource-poor context. 

In the final phase, we consulted literature related to potential modifications to a given intervention that would better suit a GBV-affected population in a humanitarian context when available. Our search strategy was informed by published materials identified in the first two phases, broadened to include work that specifically identified modifications, practical changes, adaptations, or other changes that lead to a significant difference in clinical efficacy when working with GBV-affected women. 

An initial draft of the findings based on the above reviews was reviewed by co-authors ER and CGM, along with invited collaborators with expertise in both the GBV-prevention and mental health fields. This helped to both identify novel articles and resources, and to further develop content into a viable reference for both GBV and mental health services-focused audiences. 

## 3. Results: Psychological Intervention Modalities

The results of the literature review and expert consultation are presented below, grouped by primary intervention modality. We identified eight potential interventions based on the three phases of review. We have presented a brief overview of the basic tenets of a given intervention, followed by evidence of efficacy and implementation considerations. We have also provided a table summarizing the findings (Table 1) as a reference for practitioners to see the benefits of these interventions side by side. 

### 3.1. Cognitive Behavioral Therapy (CBT) and CBT-Based Techniques 

Cognitive behavioral therapy (CBT) has been the dominant approach to psychotherapy in both research and practice for decades [17]. Its core tenets are identifiable across many interventions that show promise when working with survivors of GBV—delineating between thoughts and feelings, challenging maladaptive cognitions, and developing healthier behavior patterns that support psychological well-being. However, calls for plurality in treatment orientation [18], and increased interest in flexible interventions that can address multiple problems [19] have led to many potential responses seeking to improve mental well-being through mental health programming in a more scalable way. Table 1 summarizes key principles, implementation considerations and evidence summaries for each of the therapeutic modalities for which evidence is available. 

Cognitive processing therapy (CPT) was developed specifically for survivors of sexual violence, and is typically used to address symptoms of PTS, though it has some efficacy in reducing symptoms of other common mental health problems such as depression and anxiety [20]. The findings of two meta-analyses [21,22] have indicated that CPT may be the most effective treatment for PTS symptoms (compared to other psychotherapies and medications) across several traumatized groups. While originally designed to address symptoms of PTS among survivors of sexual violence, CPT has shown effectiveness among other trauma-exposed populations via different modalities (e.g., combat veterans via teletherapy [23] and active duty military clients in group contexts [24]). Session content for CPT mirrors typical cognitive behavioral therapy. The activities are all built on challenging the client’s appraisal of traumatic events to shed light on a healthier narrative.

In a trial conducted among sexual violence survivors in the Democratic Republic of Congo, there was a significant reduction in depression and PTS symptoms within the treatment group, as well as decreased stigma regarding sexual violence [25,26]. Recent results currently under review suggest that those symptom reductions have persisted beyond the initial trial—in a four-year follow-up, the same participants reported both lower depression and PTS scores, as well as specific knowledge and implementation of CPT skills they had learned from their therapy sessions four years prior. Participants continued to practice CPT skills to alleviate psychological distress even though CPT groups were no longer officially meeting. In a qualitative investigation from the same four-year follow-up study [27], practitioners retained CPT skills and maintained CPT groups with new clients despite a lack of institutional support and clinical oversight, indicating some degree of sustainability with limited resources. While CPT can be delivered in group or individual settings by trained psychotherapists, paraprofessionals, or lay providers, there is little evidence to date regarding its integration into extant GBV or health service infrastructure.

The common elements treatment approach (CETA) encompasses elements of several cognitive and behavioral therapies. The CETA is intended to be transdiagnostic—it does not explicitly address the symptoms of a single diagnosis but seeks to address psychological distress and well-being more broadly, independent of potential diagnoses. The CETA can address symptoms of depression, anxiety, and PTS. While modular therapies were developed in the United States [28,29], the CETA itself was specifically designed for use in low-income and humanitarian contexts. Modules were developed based on systematic reviews and meta-analyses of numerous therapies, and composed based on expert consultation and peer review [30]. Depending on symptom scores across three domains (i.e., anxiety, depression, and PTS), different modules related to each specific problem area can be included and reordered to better suit the pressing needs of an individual client. At twelve sessions, it is somewhat time intensive, though a shorter, five-session version is currently being evaluated among conflict-affected veterans in Ukraine [31].

RCTs for the CETA conducted in Thailand [32], Zambia [33], Iraq [34], Iraqi Kurdistan [35], Colombia [36], and Somalia [37] have all indicated significant positive effects on mental health outcomes for men, women and children. There is evidence for effectively addressing the incidence of GBV after incorporating safety planning and substance use programming for partners concurrently receiving the CETA, which in turn suggests that mental health programming alone may reduce the likelihood of IPV [33,38]. In a trial in Zambia, a CBT-oriented substance use component was introduced for men, while women and family members received session content related to substance support content to help facilitate discussion of drinking, its triggers, and problem behaviors more effectively. Additional safety planning and support were also included as part of the original safety module. Trials have been conducted across a variety of delivery settings, from homes to clinics to refugee camps, suggesting potential for adaptation to multiple contexts. The CETA is perhaps most resource intensive in its training and implementation. Providers must undergo at least two weeks of intensive training by certified trainers of trainers (TOTs), who in turn provide remote clinical supervision and booster training. To date, the CETA has not been integrated into extant aid programming (e.g., anti-poverty programming or GBV-specific programming) and has been typically implemented as a standalone intervention with its own infrastructure.

Interpersonal psychotherapy (IPT) is considered a benchmark approach to treating depression. IPT is rooted in CBT but incorporates attachment theory and contemporary psychodynamic theory to focus specifically on how a client’s relationship to others can influence well-being. IPT begins by identifying one of four potential problem areas that is contributing to psychological distress: interpersonal disputes, role transitions, grief and loss, and interpersonal sensitivity [39]. These factors represent a triggering point that, in conjunction with the biopsychosocial elements unique to the individual, results in distress. Though IPT is focused on addressing depression symptoms, successful trials in the developing world (e.g., Bolton et al., 2003) have relied on extensive qualitative research and adaptation to include both idiomatic expressions of mental health symptoms unique to a given context. 

One of the first landmark trials of a mental health intervention in a low-income context examined the effect of group IPT provided by a trained lay provider among men and women in rural Uganda [40]. The results demonstrated reduced depression symptoms and improved daily functioning (i.e., the ability to perform tasks required for daily living) among group participants. A pilot trial in Egypt among Sudanese refugees reported decreased depression and PTS symptoms among its participants [41]. There is additional evidence that supports efficacy among GBV survivors—an RCT in Kenya among HIV-positive women affected by GBV reported support for both the content and structure of a group IPT intervention, as well as its feasibility [42,43]. Given its effectiveness in treating depression, the WHO has released an adapted and translated manual for group IPT, and recommend it as a “first-line” treatment for depression [44]. While shorter versions have been manualized and tested in the US primary care setting (see Interpersonal Counseling [45]), IPT can be intensive, covering 16, 90 min sessions in the full-format version.

Problem management plus (PM+) is a recent development within the field of interventions for low-income settings, including humanitarian settings. PM+ was conceptualized by the WHO as part of its scalable interventions initiative [46]. PM+ is transdiagnostic, and incorporates strategies related to problem solving and behavioral therapies to address several domains related to psychological well-being—managing stress, managing problems, behavioral activation, and strengthening social support. PM+ is intended to meet task-shifting requirements in settings where more intensive psychotherapy might not be readily available. As such, it is being tested in low-income settings. Given its relative novelty, the evidence base for PM+ is still in development. A subgroup analysis from a fully-powered RCT in Kenya specifically examining GBV-affected women indicated moderate reductions in general psychological distress after a 3 month follow-up [47], while an RCT among women in conflict-affected rural Pakistan demonstrated significant reductions in depression and anxiety symptoms [48]. Pilot findings from the same trial indicated positive findings regarding the intervention’s feasibility, uptake, and acceptance by participants [49]. While GBV survivors have been included in subgroup analyses during initial testing, there are no specific recommendations for use when working specifically among GBV-affected groups. 

PM+ has potential to be a highly scalable intervention platform for use by non-specialists and specialists alike. There are existing manuals that have been adapted and translated into multiple languages available. At five 90 min sessions, it is among the shortest of interventions de-scribed. While individual treatment has been tested in two RCTs, a group adaptation is currently in development and being tested [50], and will consist of five three-hour long sessions of eight participants per facilitator. PM+ is not designed for severe mental health problems and is intended to be used with individuals with depression, anxiety, or PTS symptoms of moderate severity who require additional support beyond what is available in a given community. 

### 3.2. Third-Wave Cognitive and Mindfulness-Oriented Therapy

Acceptance and commitment therapy (ACT) incorporates elements of cognitive therapies and mindfulness-based activities to address mental health symptoms. The evidence base for ACT is still growing, but early studies and an updated meta-analysis of clinical trials indicates that it is efficacious in treating multiple psychological problems including depression, anxiety, and PTS symptoms [51]. ACT is unique in its emphasis on cultivating present-mindedness—using techniques to emotionally and cognitively “ground” one’s self in the present moment—to address mental health problems and distress. ACT is focused on identifying, clarifying, and ultimately enacting a client’s values [52]. These values are client-selected components of life that the client finds rewarding, reinforcing, and ultimately critical for well-being. Its emphasis is less on attempting to remove psychological distress than on acknowledging its existence, and identifying ways to safely and constructively work around it [52]. As a transdiagnostic approach, ACT is potentially well suited for broad-based service platforms (e.g., community-level interventions) but more research is needed. There is evidence to support the use of ACT and ACT-based interventions in both group and individual applications, with both trained lay providers and psychological professionals. ACT is not session limited or structured, and as such does not have a fixed implementation timeline, which may require additional adaptation on behalf of the provider. 

The evidence for ACT’s efficacy in humanitarian settings is limited, but growing. A cadre of counselors in Sierra Leone were recently trained in ACT with high fidelity and uptake, which suggests feasibility in scaling up services conducted by paraprofessionals [53]. The World Health Organization (WHO) developed an ACT-based guided self-help intervention as part of its scalable psychotherapy initiative [46], Self-Help Plus (SH+) [54]. SH+ relies on a printed guidebook and audiovisual sessions to provide ACT-based coping skills for large groups (20–25 participants) experiencing mild to moderate psychological distress. In an RCT [55] among South Sudanese refugee women, SH+ was associated with moderate reductions in general psychological distress, depression, and PTS symptoms. Within the sample, 26% of women reported IPV, 10% reported sexual violence, and 7% reported sexual violence by someone other than their partner.

Contemporary research is beginning to focus more on the potential for yoga, body-oriented, and mindfulness-based approaches to address negative mental health symptoms among survivors of GBV. There is a notable link between the experience of traumatic stress and biophysical/neurological problems [56,57], and meditation/mindfulness-based interventions are centered around that link. The goal of mindfulness-oriented interventions for psychological distress is to increase personal insight and improve self-referential processing [58], or one’s ability to understand and process emotions in a meaningful way. For example, trauma-sensitive yoga (TSY) combines physical poses, focused breathing, and mindfulness practice as an intervention for traumatic stress [59]. In one RCT, women with chronic, treatment-resistant PTS problems who completed a 10 week TSY program experienced a significant reduction in PTS symptoms that were sustained for a greater length of time compared to women who received only conventional talk therapy [60]. In a follow-up study among the same participants, women who continued their TSY practice had greater likelihoods of lower PTS symptoms at reassessment [61]. While clinical evidence is limited, these findings suggest the potential longitudinal effects of sustained TSY practice on mitigating trauma symptoms. Feasibility and case studies [62,63] show that TSY is a promising intervention specifically for survivors of intimate partner violence, but there are no robust investigations among that population. Similarly, there is little to no available research examining TSY in humanitarian or low-income settings, though one small study in Uganda shows promising results [64]. TSY sessions are conducted by trained, certified instructors, which may limit its feasibility in certain contexts. However, the premise of combining light physical activity, breathing, and mindfulness activities could potentially be implemented through trained lay providers. 

### 3.3. Exposure-Oriented Interventions

Eye movement desensitization and reprocessing (EMDR) was conceptualized as a safer therapeutic approach to imagined exposure therapy for traumatic events. EMDR posits that negative thoughts, feelings, emotions, and behaviors are the result of lingering memories of a potentially traumatic event. The primary theory, the adaptive information processing hypothesis, suggests that the process of repetitive, side-to-side eye movement triggers a cognitive state that ultimately facilitates information processing. EMDR has been considered a highly effective treatment for PTS symptoms, including among survivors of sexual violence [65], though recent meta-analysis findings suggest that it is not effective for addressing other problems long term [66].

EMDR has limited testing in humanitarian contexts, with mixed findings. A trial conducted with Syrian refugees [67] indicated mild symptom improvement, but reported issues with treatment fidelity and attrition. Successful training of psychological professionals has been reported in the Arab world [68], as well as natural disaster-affected and humanitarian settings in Asia [69]. No studies have been conducted in an international context specifically among GBV-affected groups, though the intervention itself was developed specifically for survivors of traumatic events. EMDR has been endorsed by the WHO’s violence against women guidelines as a potential intervention to address mental health problems stemming from exposure to sexual violence [70]. 

While there is a substantial number of studies that indicate EMDR’s potential for treating symptoms of PTS, a recent meta-analysis suggests a high risk of bias across many of the studies included, and relatively small effect sizes for treatment efficacy [66]. Moreover, the study indicated limited evidence for reducing comorbid symptom severity among several common mental health problems including depression, anxiety, and sub-stance use problems. No studies to date have examined EMDR’s potential as a group intervention, or with therapy conducted by lay providers in humanitarian contexts. As such, it may require intensive training, clinical supervision, and access to psychological professionals for implementation, or considerable adaptation for task-shifting approaches, and is therefore unlikely to have broad applicability in resource-poor settings with limited numbers of trained professionals. 

Narrative exposure therapy (NET) is a short-term intervention that draws on a variety of disciplines. NET has been used effectively with children and adults across a variety of settings, including refugee and humanitarian contexts [71,72,73]. NET is designed to be used in low-resource contexts and relies on qualitative and anthropologically oriented techniques to place trauma and related distress in cultural context. NET has been used across multiple humanitarian contexts, including in some of the earliest trials of MHPSS interventions in low-income settings [74,75]. Meta-analysis data from several studies assessing NET administered by trained lay providers in refugee settings have shown moderate effect sizes for the treatment of PTS [72]. An RCT of female former child soldiers in the DRC demonstrated the efficacy of a group version of NET in reducing PTS, depression symptoms, and aggressive behaviors in the midst of ongoing conflict [76]. No specific modifications have been identified for working with GBV survivors. As a trauma-focused intervention, NET accommodates a range of potentially traumatic events. 

The trials cited above provide evidence for the efficacy of both group and individual versions of NET. Given its emphasis on embracing culture and context, NET prioritizes training local partners and paraprofessionals as providers, though psychology professionals could also be trained. NET consists of ten 60 to 90 min sessions and requires approximately ten days of training for facilitators. Given its reliance on text and writing, it may not be well suited for low-literacy populations. However, alternative practices to accommodate clients are available (e.g., relying on art, photography, or spoken word).

**Table 1:** Summary of therapies, principles and implementation considerations for identified mental health interventions.

Intervention Name	Conventional Use and Principles	Use in Humanitarian Settings	Special Considerations for GBV-Affected Clients?	Necessary Resources/Implementation Issues
Acceptance and Commitment Therapy (ACT)	Newer cognitive therapy with explicit focus on mindfulness-based activities and practices. Transdiagnostic—not designed to address any specific mental health problems but to address symptoms of psychological distress. ACT emphasizes present-moment awareness and uses skills training to teach clients how to connect with the present (e.g., noticing physical sensations and breathing). The primary focus of ACT sessions is identifying a client’s values—client-selected features of a client’s life that are rewarding, reinforcing, and critical for well-being. ACT sessions attempt to clarify those values, and devise strategies and activities a client can pursue that strengthen commitment to those values. Goal is to improve ‘psychological flexibility’—the ability to navigate negative experiences with openness and awareness.	The World Health Organization (WHO) developed, piloted, and tested an ACT-based intervention specifically for use in humanitarian settings (Self-Help Plus). More than 60% of the participants—South Sudanese refugee women in Northern Uganda—reported some form of gender-based violence. Results indicated a significant reduction in general psychological distress, as well as depression, PTS and anxiety symptoms. Successful training of ACT counselors in Sierra Leone.	ACT-based programming for GBV survivors may specifically emphasize issues of experiential avoidance [77], e.g., using present-moment awareness skills when walking past the site of a sexual assault to mitigate psychological distress, decrease avoidance behaviors, and promote positive coping strategies for negative thoughts/feelings. Possible risk of respondents accepting abuse as inevitable. Primer for facilitators that highlights the risks of working with GBV-affected groups is strongly recommended.	Transdiagnostic approach suits more broad-based service platforms, but evidence base is limited. Group or individual applications possible, with lay and professional providers. Training can be intensive (two or more weeks) or simplified (one week) depending on session content. No inherent session count but some interventions such as SH+ rely on five two-hour sessions in groups of 20 or more participants, in addition to a manual and audio sessions. Audio sessions and manual are available in multiple languages online.
Cognitive Processing Therapy (CPT)	Developed for survivors of sexual violence in the US. Use among many trauma-affected populations (e.g., combat veterans and rape survivors). Most commonly used to address PTS symptoms, but can treat comorbid depression and anxiety. Very similar to cognitive behavioral therapy (CBT) Uses Socratic dialogue to address “Stuck Points”—extreme, exaggerated, often negative statements about self or others. Special focus on issues of power, control, esteem, intimacy, and safety.	Survivors of sexual violence in rural Eastern DRC saw reduced depression and PTS symptoms. ○ Evidence of sustained impact on symptoms and skill retention at four year follow-up. Reduced depression and functional impairment among conflict-exposed men and women in Iraqi Kurdistan. Reduced depression and PTS symptoms among men and women exposed to conflict in southern Iraq.	This therapy was designed specifically for use with sexual violence-affected populations, but can be modified to include more specific considerations such as ○ Safety planning for women experiencing intimate partner violence (IPV). ○ Addressing hypervigilance to accurately assess current risk for IPV. ○ Co-occurring case management.	CPT manuals available for group and individual programs. Evidence for both professional and lay providers as effective facilitators. Does not require additional technology or infrastructure, but relies on extensive training and supervision. Complex intervention with detailed, intensive content might be difficult for scaling. Specialized for treating trauma-exposed groups—highly focused on traumatic events. Training process is approximately two weeks, with booster sessions. Twelve, one-hour sessions can be demanding in conflict settings but reduced/modified manuals are validated and available. Weekly clinical supervision and annual skills booster trainings recommended. Handouts and homework assignments may be problematic for populations with low literacy. Specialized intervention that may not be suited for more community-oriented MHPSS programming, e.g., child-friendly spaces.
Common Elements Treatment Approach (CETA)	The CETA is based on research related to modular therapies [28,29]—MHPSS programs with different structures and content depending on the symptoms described by the client. Modules or elements were developed based on aggregated components of evidence-based therapies. Sessions follow a typical CBT structure, with pre-described, manualized content for each session depending on the primary presenting problem—anxiety, depression, PTS, or combinations of each. Content focuses on various aspects from multiple evidence-based interventions such as psychoeducation, behavioral activation (getting active), in vivo exposure, cognitive restructuring (thinking in a different way), or safety planning.	Multiple successful trials for common mental health problems across several humanitarian and low-income contexts, including refugees, conflict-exposed populations, and other high-risk and vulnerable groups in Sub-Saharan Africa, Eastern Europe, South East Asia, South America, and the Middle East [32,37].	Recent clinical trial data among families living in Lusaka, Zambia tested the CETA with the specific goal of reducing IPV and alcohol abuse [33]. ○ Results indicate a significant reduction in IPV. experienced by women ○ Lower alcohol abuse from men. A CBT-based substance use component was included for men, and a substance support component was included for women to help partners discuss problem drinking and its triggers more openly/effectively, as well as safety monitoring check-ins and planning [38].	The CETA has a diverse, established literature base demonstrating evidence for both individual and group therapy, conducted by lay and professional facilitators. It is transdiagnostic, and is capable of addressing multiple mental health problems with specific modular sessions. Typically twelve sessions, though shorter versions are being tested currently in Ukraine. Has been delivered successfully in diverse settings from homes to clinics to community-based organization offices. Training is time intensive, and relies on specialized consultation with existing trainers of trainers. Additional booster training and clinical supervision is also required for providers. No current evidence of use as an integrated MHPSS intervention with other areas (e.g., GBV specific or poverty alleviation); typically used as a standalone intervention with its own infrastructure. Must be purchased.
Eye Movement Desensitization and Reprocessing (EMDR)	Treatment specifically for PTS symptoms that asks clients to recall unpleasant or traumatic memories while making. horizontal eye movements [78]. Considered a highly effective treatment for PTS in short term, but no evidence of consistent change for other problems [66]. The theory of change for EMDR is unclear but is typically explained using the adaptive information processing hypothesis; that the process of eye movement triggers a state that facilitates information processing and allows clients to overcome traumatic experiences safely.	Evidence is a bit mixed—small trial among Syrian refugees reported mild improvements despite fidelity and attrition problems [67]. Similar findings have been reported in the conflict-affected parts of the Arab world [68], and natural disaster-affected and humanitarian settings in Asia [69], though the studies involved are of mixed quality. No studies specifically addressing GBV-affected populations.	Like other therapies listed here, EMDR was developed specifically for trauma survivors, including women exposed to GBV and sexual violence. No specific adaptations to address GBV.	Evidence base shows consistent effectiveness across variety of contexts, but recent meta-analysis indicates a high risk of bias across many studies and small effect sizes [66]. No studies to date have examined group sessions or therapy conducted by lay providers, which may limit feasibility in certain contexts. Highly specialized intervention specifically for traumatic stress, with limited evidence of reducing comorbid mental health symptoms. Manuals/training must be purchased.
Interpersonal Psychotherapy (IPT)	Developed in the US as a dedicated intervention for major depressive disorder [79]. Strong cognitive roots, but focuses more on relationships between client and environment/others. Basic premise is that how we relate to others can drive psychological well-being. Treatment is based on the identification of one of four problem areas: interpersonal disputes, role transitions, grief and loss, and interpersonal sensitivity [39]. Therapist uses communication analysis—a way to investigate how interpersonal difficulties are linked to expression/communication.	One of the first trials of an MHPSS intervention in humanitarian settings involved using group IPT among men and women in Uganda [40]. Significant effects for reduced depression and improved daily functioning. A pilot trial in Cairo, Egypt among Sudanese refugee men and women noted decreased depression and PTS symptoms [41]. Work in Kenya [42] among HIV+ women, many of whom were affected by GBV, reported support for both group IPT and its feasibility.	Dedicated depression intervention with mixed evidence for other common mental health problems. Limited research looking specifically at the efficacy of IPT within GBV-affected populations—one study developed a specific manual for working with survivors of sexual violence [80]. Emphasis on interpersonal relationships could be beneficial for addressing depression and anxiety associated with GBV exposure, though limited evidence and few trials.	Group version manualized by the WHO as part of mhGAP programming. Effective when conducted by both lay and professional providers. Full format is intensive; typically contains sixteen weekly 90 min sessions. Five-session version is available and manualized (interpersonal counseling). Highly specialized, with demonstrated efficacy in addressing depression; may not be suitable for community-oriented programming. Depression focused, but content can be adapted to focus on idiomatic expressions of symptoms. Requires clinical supervision and oversight, especially when relying on lay providers.
Narrative Exposure Therapy (NET)	NET is a short-term, trauma-specific intervention developed to address PTS symptoms exposed to violent trauma. Therapy sessions are devoted to the construction of a single, coherent trauma narrative from all the disparate pieces of the event, i.e., an often-written testimonial account of the sensory, psychological, and cognitive experiences of the trauma incident. Begins with symptom assessment and continues with the development of the cohesive trauma narrative building in each session. Experiential session content can draw from art therapy and other self-expressive techniques.	Some of the earliest trials for mental health programming in humanitarian contexts tested NET [74,75]. Randomized trial of female former child soldiers in DRC shows efficacy of group NET among girls exposed to sexual violence in reducing PTS, depression, and aggressive behaviors despite ongoing violence [76]. Some evidence from small-sample work with survivors of human trafficking [81].	Developed for addressing trauma, there are no specific guidelines or strategies for working with GBV-affected groups. Working in groups can present issues of stigma. Effective as both a group and individual therapy, with specific guidelines on how to address deeply personal trauma with others in a supportive fashion. While not specifically developed for GBV-affected populations, it focuses specifically on trauma, and GBV/sexual violence are often motivating examples in treatment guidelines.	Can be conducted in groups or individually. Evidence supports both professional and trained lay facilitators. Moderate intensity regarding session count—ten 60 to 90 min sessions. Ten days or fewer needed for training. May not be well suited for low-literacy populations given emphasis on written statement, but alternatives (e.g., art, photography, and spoken word) may potentially be used. Can be more readily integrated into extant community-based programming, e.g., clinics and parenting classes. Manuals/training must be purchased.
Problem Management Plus (PM+)	Conceptualized by the WHO as part of its low-intensity psychotherapy initiative to develop MHPSS programs that require fewer resources. Transdiagnostic approach that incorporates problem-solving and behavioral strategies to address four domains: managing stress, managing problems, behavioral activation, and strengthening social support [82].	Relatively new therapy; as such, evidence is just starting to come out. Randomized trial in Kenya with women demonstrated moderate reductions in general psychological distress among GBV-affected women after a 3 month follow-up [47]. Pilot trial in Pakistan among women in a conflict-affected rural area demonstrated positive findings regarding the intervention’s feasibility, uptake, and acceptance by participants [49]. The full trial indicated a significant reduction in depression and anxiety symptoms [48].	No specific adaptations recommended or assessed when working specifically with GBV survivors. Lack of specific symptom focus allows for greater breadth when discussing problems unique to GBV-affected groups. The current manual provides guidelines for working with survivors of sexual violence, including recommendations for safety planning and additional sensitivity when working with affected clients ○ Discussing stigma, local taboos or cultural difference, and familial rejection.	Low-intensity package intended to be delivered by non-specialist, easily trainable and scalable. Broad-based, and designed to address many different symptoms of common mental health problems (i.e., transdiagnostic). Manualized for free; available in multiple languages. Easily adapted to novel settings, with existing manuals in multiple languages. Five 90 min sessions of individual therapy. Group version in testing now [50] with three hour sessions with 8 participants. Not suitable for severe mental health problems. Does not provide trauma-specific guidelines for care.
Trauma-Sensitive Yoga (TSY)	Research-supported link between psychological trauma, neurological problems, and physical sensations [56,57]. Incorporates physical poses, breathing, and mindfulness practice to reduce symptoms of psychological distress and trauma.	Limited research regarding efficacy in low-income and humanitarian contexts. Some research to indicate positive outcomes from program in Uganda [64] based on small trial and pilot work. Findings from small studies in high-income contexts demonstrate TSY is a promising intervention specifically for survivors of intimate partner violence, but there are no robust investigations among that population [62,63].	Developed to address biophysical and mental health-related trauma symptoms. Special considerations and modifications can be made to accommodate injuries or physical pain caused by GBV.	TSY sessions require instructors to be trained and certified in the practice, limiting feasibility in humanitarian settings. Combining light physical activity, breathing, mindfulness activities as either group or individual-based programs could potentially be implemented through trained lay providers.

## 4. Discussion

This review provides a brief review related to specific psychological interventions for addressing mental health problems among survivors of IPV or sexual violence in humanitarian settings, as well as specific considerations for working within those contexts. Transdiagnostic treatments that can be delivered by paraprofessionals or trained lay providers have the potential to reach many survivors in need, and have been shown to reduce mental health symptoms, as well as general psychological distress. 

Contemporary research is moving towards a less medicalized approach to mental health, a greater emphasis on transdiagnostic, broad-based approaches with greater scalability, i.e., programs that avoid building entirely new infrastructures to address single mental health diagnoses. Contemporary practice is looking more to integrating mental health/psychological interventions into broader social intervention programs, such as incorporating mental health-oriented programming into violence prevention or poverty alleviation initiatives. In addition, mental health providers/services need to better integrate violence prevention given the prevalence of GBV and its association with mental health problems. Highly specialized mental health programming is not a panacea for addressing psychological well-being in complex emergencies. Adapting specialized mental health treatments with a dedicated diagnostic focus to make them more scalable and more easily implemented as community and family supports should be the focus of future programming. 

Future research may seek to demonstrate the potential for broad-based mental health programming as a preventative intervention, e.g., integrating basic psychological screening and stress management skills training into primary care or community centers in an effort to prevent development of more severe symptoms. There is some evidence that psychological/psychosocial interventions can contribute to reductions in violence [12,14].

There is a notable gap in that many of these studies fail to include men in trials, despite evidence that psychological distress in men is associated with an increased likelihood of living in poverty, abusing alcohol, and perpetrating partner violence [83,84]. Evidence suggests that addressing men’s mental health and substance issues may decrease the likelihood of violence perpetration [33]. Research in this area might provide insight into strategies that mitigate both violence and poor mental health. Studies conducted among men in the several low-income and humanitarian settings suggest a link between men’s exposure to violence in early adulthood and an increased likelihood of perpetrating IPV [85,86]. The same study indicated that men typically seek psychological coping strategies that reaffirm heteronormative gender expectations of male dominance, including alcohol use, physical/psychological abuse, and abandoning romantic partners who have experienced sexual violence [85]. As such, mental health approaches that simultaneously address life course exposure to potentially traumatic events, psychological distress, and broader sociocultural issues concerning masculinity and power may prove more effective in reducing GBV. Similarly, concurrent interventions that can address substance and alcohol abuse, such as motivational interviewing, may increase the likelihood of finding healthier coping strategies and reducing violence.

This study is presented with some limitations. While the authors did follow a standardized approach to identifying interventions in the literature, exploring their principles, and examining the extent to which they would be feasible in humanitarian context, this is not a systematic review. As such, it is not a comprehensive evaluation of the overall status of the field, or of broader therapeutic efficacy within these circumstances; however, it does provide valuable insights into the extent to which these interventions are adaptable to GBV or humanitarian settings and adjustments necessary for successful implementation. 

## 5. Conclusions

Although this review does not rely on the conventional systematic review approach, we believe that a review with a more programmatic/clinical perspective on identifying and implementing evidence-based mental health programming will be useful to mental health practitioners and GBV advocates and practitioners to understand what evidence is available for different types of psychological interventions. Ultimately, these interventions and their associated research are critical steps in advancing mental health programming to a point where it can be safely integrated into established programming in a less intensive, more community focused way that remains beneficial to communities affected by GBV.

## Appendix A. Search Terms Included in Phase 1

“Psychotherapy” [Mesh] OR “Psychotherap*” [tw] OR”Counseling”[Mesh]OR “Counselling”[tw]OR”Behavior Therapy”[Mesh]OR“Behaviour Therapy”[tw] OR“Behavioral Therapy”[tw] OR “BehaviouralTherapy”[tw] OR “Behavioral Management”[tw] OR “Behavioural Management”[tw] OR”Cognitive Therapy”[Mesh] OR “Cognitive Behavior Therapy”[tw] OR “Cognitive Behaviour Therapy”[tw] OR“Cognitive Processing Therapy”[tw] OR“Interpersonal Therapy”[tw]OR “Interpersonal Psychotherapy”OR“Prolonged Exposure”[tw] OR“Prolonged Exposure Therapy”[tw] OR “Narrative Exposure Therapy”[tw] OR “Eye Movement Desensitization and Reprocessing”[tw] OR “Behavioral Activation”[tw] OR “Behavioural Activation”[tw]OR “Evidence-Based Practice”[Mesh] OR “Evidence-Based Therapy”[tw] OR “Evidence-Based Therapies”[tw]

And

“Mental Disorder” [Mesh] OR “Mental Disorder*”[tw] OR “Common Mental Disorder*”[tw]OR “Mental Health”[tw] OR “Symptom”[tw] OR “Symptoms”[tw] OR“Distress”[tw] OR”Anxiety”[tw] OR “Anxiety Disorder”[tw]OR “Anxiety Disorders”[tw]OR “Agoraphobia”[tw] OR “Obsessive-Compulsive Disorder”[tw] OR “Panic Disorder” OR“Phobic Disorder”[tw] OR “Phobia”[tw] OR “Phobias”[tw]OR “Mood Disorder”[tw]OR “Mood Disorders”[tw]OR“Affective Disorder”[tw] OR“Affective Disorders”[tw] OR “Depress*”[tw]OR “Depression”[Mesh]OR “Depressive Disorder”[tw]OR “Depressive Disorders”[tw]OR “Major Depression”[tw]OR“Major Depressive Disorder”[tw] OR“Stress”[tw] OR “Trauma”[tw] OR“Traumas”[tw] OR”Psychological Trauma”[tw]OR“Psychological Traumas”[tw] OR”Trauma and Stressor Related Disorder”[tw] OR Trauma and Stressor Related Disorders”[tw] OR”Stress Disorder”[tw] OR”Stress Disorders”[tw] OR“Post-TraumaticStress”[tw] OR “Post-Traumatic Stress Disorder”[tw]OR “Acute Stress Disorder”[tw] OR” AcuteTraumatic Stress Disorder”[tw]

And

“Algeria” OR “Angola” OR “Bangladesh” OR “Belize” OR “Benin” OR “Bhutan” OR “Bolivia” OR “Cabo Verde” OR “Cambodia” OR “Cameroon” OR “Comoros” OR “Congo” OR “Cote d’Ivoire” OR “Djibouti” OR “Egypt” OR “El Salvador” OR “Ghana” OR “Haiti” OR “Honduras” OR “India” OR “Indonesia” OR “Iran” OR “Kenya” OR “Kiribati” OR “Kyrgyz*” OR “Lao” OR “Lesotho” OR “Mauritania” OR “Micronesia” OR ”Mongolia” OR “Morocco” OR “Myanmar” OR “Nepal” OR “Nicaragua” “Nigeria” OR “Pakistan” OR “Papua New Guinea” OR “Philippines” OR “Samoa” OR “Sao Tome Principe” OR “Senegal” OR “Solomon Islands” OR “Sri Lanka” OR “Tajikistan” OR “Tanzania” OR “Timor-Leste” OR “Tunisia” OR “Ukraine” OR “Uzbekistan” OR “Vanuatu” OR “Vietnam” OR “West Bank” OR “Gaza” OR “Zambia” OR “Zimbabwe” OR “Afghanistan” OR “Burkina Faso” OR “Burundi” OR “Central African Republic” OR “Chad” OR “Eritrea” OR “Ethiopia” OR “Gambia” OR “Guinea” OR “Guinea-Bissau” OR “Liberia” OR “Madagascar” OR “Malawi” OR “Mali” “Mozambique” OR “Niger” OR “Rwanda” OR “Sierra Leone” OR “Somalia” OR “South Sudan” OR OR “Sudan” OR “Syria” OR “Togo” OR “Uganda” OR “Yemen”.
